# Evaluation of the Efficacy of Commercial Disinfectants against African Swine Fever Virus

**DOI:** 10.3390/pathogens12070855

**Published:** 2023-06-21

**Authors:** Lorraine Frost, Matthew Tully, Linda Dixon, Hayley M. Hicks, Jessica Bennett, Isobel Stokes, Laura Marsella, Simon Gubbins, Carrie Batten

**Affiliations:** 1 The Pirbright Institute, Pirbright, Woking GU24 0NF, UK; matt.tully@pirbright.ac.uk (M.T.); linda.dixon@pirbright.ac.uk (L.D.); hayley.hicks@pibright.ac.uk (H.M.H.); jessica.bennett@pirbright.ac.uk (J.B.); is00330@surrey.ac.uk (I.S.); laura.marsella@syngenta.com (L.M.); simon.gubbins@pirbright.ac.uk (S.G.); carrie.batten@pirbright.ac.uk (C.B.); 2 Department of Microbial Sciences, School of Biosciences, Faculty of Health and Medical Sciences, Stag Hill Campus, University of Surrey, Guildford GU2 7XH, UK; 3 Jealott’s Hill International Research Centre, Syngenta, Bracknell RG42 6EY, UK

**Keywords:** African swine fever virus, disinfectant, inactivation, control

## Abstract

African swine fever (ASF) is an economically important disease due to high morbidity and mortality rates and the ability to affect all ages and breeds of pigs. Biosecurity measures to prevent the spread of the causative agent, African swine fever virus (ASFV), include prescriptive cleaning and disinfection procedures. The aim of this study was to establish the biocidal effects of twenty-four commercially available disinfectants including oxidizing agents, acids, aldehydes, formic acids, phenol, and mixed-class chemistries against ASFV. The products were prepared according to the manufacturer’s instructions and a suspension assay was performed with ASFV strain, BA71V using Vero cells (African green monkey cells) to test efficacy in reducing ASFV infection of cells. Generally, disinfectants containing formic acid and phenolic compounds, as well as oxidizing agents reduced viral titers of ASFV by over 4 log_10_ at temperatures ranging from 4 °C to 20 °C. Hydrogen peroxide, aldehyde, and quaternary ammonium compounds containing disinfectants were cytotoxic, limiting the detection of viral infectivity reductions to less than 4 log_10_. These preliminary results can be used to target research on disinfectants which contain active ingredients with known efficacy against ASFV under conditions recommended for the country where their use will be applied.

## 1. Introduction

African swine fever (ASF) is a hemorrhagic disease affecting domestic and wild pigs. The disease is caused by the African swine fever virus (ASFV); an enveloped, large DNA virus, the only known member of the family *Asfaviridae* [1]. Highly virulent strains can cause disease with morbidity and mortality rates near 100%. ASF is endemic in sub-Saharan Africa and Sardinia with the most recent incursion beginning in Georgia in 2007 which led to unprecedented spread [2]. Between 2007 and 2012, ASF spread through Armenia, Azerbaijan, and Russia before spreading into mainland Europe. In mid-2018, ASF entered China and spread throughout the region [3,4]. In July 2021, ASFV was reported in the Dominican Republic, signifying the first outbreak in the Americas in over 40 years [5]. The disease has now been reported in 45 countries including EU member states, parts of Asia, Oceania, and the Americas [6]. Since there is no suitable ASFV vaccine, control of ASF is supported through stamping out in affected holdings, cleaning, and disinfection and the application of biosecurity measures. Globally, millions of pigs destined for the pork market have been destroyed resulting in huge economic losses. China, the world’s biggest producer of pork products, has estimated economic losses between 2018 and 2019 to be USD 111.2 billion [7]. Of these losses cleaning and disinfection represented a comparatively low-level expenditure of approximately USD 55 million and represents an area where a relatively small investment could lessen the impact of ASF [7]. Fomites contaminated with blood or excretions represent a major risk of secondary infections and, therefore, some countries have developed prescribed protocols for cleaning and disinfection [8,9]. Recently, two reviews on the control of ASFV using disinfectants were published, identifying the need for more data on the virucidal efficacy of certain chemical compounds [10,11]. In 2022, in response to the incursion of ASFV into the Americas, The National Institute of Food and Agriculture (NIFA) announced a USD 5 M Investment in agricultural biosecurity program to prevent, detect, and respond to potential spread of ASFV into the USA [12].

Countries have developed different rules for approving disinfectants for use during notifiable disease outbreaks. In the USA, this is set out by the Environmental Protection Agency (EPA) detailing testing methods described in ASTM E1053 (ASTM, 2020), published by the American Society for Testing and Materials (ASTM) [13]. In the UK, a disinfectant approved by the Department for Environment, Food and Rural Affairs (Defra) must be used in the event of a notifiable disease outbreak. There are four specific disease orders: The foot-and-mouth disease order, the diseases of poultry order, the tuberculosis in animals order and the swine disease order, which specifically covers swine vesicular disease virus (SVDV). All other notifiable diseases, which include other swine diseases such as ASF are covered under the “General order” (GO) category which uses *Salmonella enterica* serovar Enteritidis NCTC 13665 as the challenge organism [14,15]. Testing differs between the two countries: the USA requires a quantitative carrier test where the virus is dried on hard non-porous surfaces, whereas the UK requires a quantitative suspension test. Additionally, the USA requires a lower pass threshold of viral titer reduction (≥3 log) compared to the UK (≥4 log) [13,15].

The Pirbright Institute performs tests on behalf of Defra for disinfectants seeking approval under the diseases of swine order (test organism, SVDV) and the foot-and-mouth disease order (test organism foot-and-mouth disease virus (FMDV)) using a suspension test [15]. An ASFV disinfectant suspension test was developed and offered commercially in 2019, in response to requests from manufacturers for evidence of efficacy against ASFV. Twenty-four commercially available disinfectants were tested at The Pirbright Institute, this study discusses these test outcomes [15].

## 2. Materials and Methods

Disinfectants within this study were tested for ASF virucidal efficacy by suspension method [16]. Test conditions including dilution of disinfectant, test temperature, and contact time were stipulated by the manufacturer.

### 2.1. Disinfectant Test

Briefly, a cell culture-adapted, non-pathogenic ASFV strain, BA71V was grown in Vero cells (African green monkey cells, ECACC 84113001) [17]. Viral samples were prepared in cells cultured in Dulbecco’s modified Eagle’s medium (DMEM) containing 1% L-glutamine and 10% heat-inactivated fetal bovine serum (FBS). Disinfectants were stored according to the manufacturer’s instructions and prepared directly before testing. Dilutions of each disinfectant were used as specified (Table 1).

An initial 10× disinfectant starting concentration was diluted in 400 parts per million (ppm) calcium carbonate to simulate hard water conditions. A total of 100 µL of each 10× disinfectant was diluted in 800 µL hard water and 100 µL BA71V stock for the time and temperature specified (Table 1). A cytotoxicity control was evaluated for each concentration of disinfectant by diluting the 100 µL of 10× disinfectant in 800 µL hard water but using 100 µL of cell culture media instead of BA71V, a neutralization control was prepared in the same way. Cytotoxicity was assessed on the integrity and appearance of the cell sheet. A neutralizer, described below, was used to arrest the virucidal activity of the disinfectant at the end of the contact time. Disinfectant activity was neutralized by serial dilution (10-fold) with either phosphate-buffered saline (PBS) containing 1% penicillin/streptomycin, 1% L-glutamine, 1% FBS, and 0.01% phenol red (used for disinfectants A, B, H, J–S and U–X) or 0.05 M carbonate bicarbonate buffer (Merck, Rahway, NJ, USA) containing 1% FBS (used for disinfectants C–G, I and T). Suppression of disinfectant activity was evaluated by adding 100 µL neutralization control to 800 µL neutralizer and then adding 100 µL positive control. Negative controls without virus or disinfectant, positive controls with virus and without disinfectant, and reference controls using a standard 0.56% formaldehyde were included in each experiment. All controls were subject to neutralization following incubation times.

### 2.2. Plaque Assay

Following treatment, a plaque assay in Vero cells was used to detect ASFV. Using confluent cell monolayers in 6-well plates, 200 µL of each serially diluted product was added in triplicate to different wells. The cells were incubated in 5% CO_2_ at 37 °C for 1 h and were overlaid with 2 mL of 1.375% Eagle’s overlay supplemented with 4% FBS, 1% Avicel solution, 0.1% penicillin/streptomycin and 0.1% L-glutamine. After incubation for 6 days, the virus titer was determined as plaque-forming units per ml (pfu mL^−1^) following staining with crystal violet solution.

### 2.3. Result Determination

Titer reductions (TR) were calculated:

TR = a − b,

where a = Titer of virus positive control and b = Titer of virus after exposure to disinfectant.

For disinfectants A, E, and V, the reduction in titer was estimated using a generalized linear model with Poisson errors and a log link function. The response variable was the number of plaques and the explanatory variable was disinfectant concentration. Model assumptions were checked by examining model residuals and calculating the variance to mean ratio, none of which suggested a substantial deviation from a Poisson distribution. Negative binomial and quasi-Poisson generalized linear models (GLMs), which relax the assumption about the relationship between mean and variance implicit in using a Poisson model, were also considered. The estimated reductions in titer were similar for all models, giving confidence that the results presented are robust.

Where no plaques were identified, titer reduction is provided as above the titer obtained in the positive control excluding the limit of detection (1.3 log_10_) and cytotoxicity.

Results were calculated using the positive control titer specific to each disinfectant test. The average titer for all positive controls used within the study was 5.24 log_10_ pfu mL^−1^ with a standard deviation of 0.50. A contact time is provided if the difference in results for the positive control and the neutralization control are ≤0.5 log_10_.

## 3. Results and Discussion

Disinfectants representing oxidizing agents, acids, aldehydes and mixed-class chemistries were tested. The most prevalent were oxidizing agents (peroxygens, iodo-phors), and mixed-class chemistries (aldehyde plus QACs, hydrogen peroxide plus peracetic acid).

One iodophor-based disinfectant was tested (A). This disinfectant reduced the titer of ASFV by over 4 log_10_ at a dilution of 1:750 within 30 min at 20 °C. A dose–response was observed, with efficacy falling above dilution 1:1500. Iodophors are generally non-toxic and their activity is not affected by hard water; however, they can stain some surfaces and can be expensive. A review of iodophor-based disinfectants recorded as approved for both SVDV and GO on the Defra-approved list indicates similar approved working dilutions for all listed iodophor disinfectants (1:100–1:150 SVDV and approx. 1:50 GO) [18]. Our results concur with previous results which showed that iodophor disinfectants have similar or greater efficacy on ASFV than SVDV [11]. There are currently no iodophor-based disinfectants listed on the “Disinfectants approved for use against African swine fever virus in farm settings” list held by the US EPA [19].

The majority of the peroxygen disinfectants submitted listed potassium peroxymonosulphate (MPS) as the main active component. Peroxygen disinfectants, except those based on hydrogen peroxide and peracetic acid, produced similar results at a dilution of 1:800 independent of temperature (4 °C, 10 °C or 20 °C). Disinfectant F inactivated over 4 log_10_ ASFV within 5 min. Disinfectant E, containing sodium percarbonate (25–50%), was tested at multiple dilutions and the results gave a dose–response. Dilutions of less than 1:3000 inactivated over 3.9 log_10_ of ASFV within 10 min at 10 °C.

There are many MPS-based disinfectants on the Defra-approved list. These disinfectants share similar approved dilution rates for FMDV (1:1200–1:1300) and also for GO (1:49–1:100). Wales and Davies determined that for one MPS containing disinfectant. ASFV is moderately less susceptible than FMDV [11]. They concluded that as the GO-approved dilutions were more concentrated than the FMDV-approved dilutions, using a MPS disinfectant at the GO dilution, would likely be effective [11]. Our data, which tested an MPS at 1:800 would at least concur with this assumption. There is currently one MPS-containing disinfectant on the US EPA-approved list and several others that list oxidizing agents as their main active ingredient.

The US EPA approved list includes one hydrogen peroxide product at a working dilution of 1:64; however, Gabbert et al., tested one hydrogen peroxide product with results indicating low efficacy, reducing ASF viral titer by less than 2 log_10_. In this study, disinfectants containing hydrogen peroxide could not be neutralized at the dilutions requested by the manufacturers and additionally were found to be cytotoxic. Therefore, additional tests would be required to form confident conclusions for contact time or effectiveness against ASFV for hydrogen peroxide-based disinfectants.

Within the aldehyde plus QAC category, two disinfectants had a basic glutaraldehyde and QAC composition whilst four incorporated additional constituents (Table 1), all were cytotoxic. Disinfectant K could not be neutralized at a dilution of 1:399. Except for disinfectant L, all products reduced the ASFV titer by over 3 log_10_ within 30 min at 10 or 20 °C. No conclusions on effective dilution could be drawn as cytotoxicity affected the maximum reportable titer reductions. Virocid, listed on the US EPA approved list contains active ingredients of aldehyde, QAC, and alcohol, most similar to product O and is approved at a concentration of 1:200 also demonstrating a greater than 3 log_10_ reduction.

One disinfectant contained only QAC, and two disinfectants listed aldehydes as active ingredients. Disinfectants Q and R were cytotoxic at 1:800 and 1:400 dilutions, respectively. Product S, containing only formaldehyde and tested at 1:1785, reduced the ASFV titer by 0.9 log_10_, lower dilutions were too cytotoxic to obtain a result.

Two disinfectants contained formic acid. Disinfectant T, tested at 10 °C and a dilution of 1:2000, reduced the ASFV titer by over 3.2 log_10_. Disinfectant U, tested at 20 °C and a dilution of 1:800 reduced the ASFV titer by over 4.2 log_10_. Lower dilution rates tested for both disinfectants were unable to be neutralized. No further data could be found for formic acid-based disinfectants and, therefore, no assumptions regarding maximum effective dilution could be made.

Finally, two phenol-based disinfectants reduced the ASFV titer by over 4 log_10_ at a dilution of 1:400 (V and W) and one reduced the titer by over 4.2 at a dilution of 1:2000 (X). Phenol-based disinfectants have a wide spectrum effect, are stable during storage, and maintain efficacy in the presence of organic matter but are toxic and corrosive.

In conclusion, decontamination including the cleaning and disinfection of premises is an important measure to halt the spread of ASFV and allow for the repopulation of previously infected premises. Disinfectants should have a wide range of use, be affordable, easy to store and prepare, and be non-toxic. Data on a range of chemistries is, therefore, required to enable an informed choice that is fit for the intended use. Knowledge gaps highlighted by Beato and co-authors listed limited data for aldehydes, with mention of glutaraldehyde, phenol and iodine compounds, and alcohols [10]. Our data suggest that iodophor and phenol-containing disinfectants are effective against ASFV. We showed that glutaraldehyde disinfectants are effective, but higher dilutions could be tested to confirm a higher than 4 log_10_ reduction in viral titer. Information in this study is preliminary and could be used by other markets to target research on disinfectants which contain active ingredients with known efficacy. This targeted research should utilize test procedures appropriate to the individual countries rules and broaden the disinfectant chemistries listed on country-specific approved lists. The study data was based on disinfectants where the manufacturers are actively seeking approval to market from their competent authority. Further studies on the disinfectant effectiveness against ASFV on different surfaces, under different conditions and potentially against different circulating ASFV strains, would benefit from being investigated.

## Figures and Tables

**Table 1:** Virucidal efficacy of 24 disinfectants against African swine fever virus under various temperatures, contact times and dilutions.

Category	Disinfectant	Active Ingredient	Temperature (°C)	Contact Time (min)	Dilution	Log Reduction
Iodophor	A	IOD (1–3%), surfactant, acid	20	30	1:750	>4.0
					1:1500	>3.1
					1:2000	1.91 (CI 1.79–2.03)
					1:3000	0.45 (CI 0.33–0.57
					1:4000	0.17 (CI 0.03–0.30)
Peroxygen (except hydrogen peroxide)	B	MPS (50%), SDIC (5%), acid, surfactant	4	10	1:800	>3.3
	C	MPS (≤50%), SDIC (≤3%), acid, surfactant, inorganic buffer	4	30	1:799	>4.5
	D	MPS (<55%), acid, surfactant, inorganic buffer	10	ND	1:800	>4.4
	E	Sodium percarbonate (25–50%), acid, surfactant, sodium salt	10	10	1:1000	>3.9
					1:3000	>3.9
					1:5000	2.41 (CI 2.06–2.78)
					1:10,000	1.97 (CI 1.64–2.31)
					1:100,000	0.14 (CI −0.20–0.48)
	F	MPS (≤100%), surfactant, alcohol	20	5	1:800	>4.2
	G	MPS (30–60%), acid, surfactant	20	30	1:800	>4.2
	H	MPS (50–100%), citric acid (2.5–10%), surfactants	20	30	1:800	>3.8
Hydrogen peroxide	I	Hydrogen peroxide (49–49.9%)	10	ND	1:7.25	>2.2 *
Hydrogen peroxide and peracetic acid	J	Hydrogen peroxide (≤22%), peracetic acid (≤4%), acids, amine oxide	21	ND	1:50	>3.2 *
Aldehyde + QAC	K	GA (≤20%), DDAC (≤10%)	4	ND	1:399	>3.6 *
	L	GA (22%), DDAC (9%), ADBAC (14.5%)	10	10	1:800	>2.2 *
	M	GA (6.25%), ADBAC (5%), DDAC (7.55%), Pine oil (2%)	10	10	1:400	>3.3 *
	N	GA (10–15%), DDAC (3–5%), surfactant, acid, alcohol	10	30	1:800	>3.4 *
	O	GA (10.7%), DDAC (8%), ADBAC (17%), alcohol	20	30	1:1500	>3.1 *
	P	GA (10–25%), ADBAC (2.5–10%), formaldehyde (10–25%)	20	30	1:400	>3.3 *
QAC	Q	DDAB (10%)	4	1	1:800	>2.3 *
			20	1	1:800	>2.4 *
Aldehyde	R	GA (9.9%), formaldehyde (9.8%), surfactant	20	30	1:400	>3.3 *
	S	Formaldehyde (39%)	20	30	1:1785	0.95 (CI 0.84–1.05)
Formic Acid	T	Formic acid (>48%), carboxylic acid, surfactant	10	30	1:2000	>3.2
	U	Formic acid (60–70%), surfactant	20	30	1:800	>4.2
Phenol compound	V	Biphenyl-2-ol (1–3%), alcohol, acids, surfactants	10	10	1:400	2.94 (CI 2.73–3.15)
				30		4.48 (CI 3.62—5.35)
	W	Mixed chlorocresols (20–40%), xylenol (1–10%), acid, alcohol, surfactant, solvent	10	10	1:400	>4.1
	X	Chlorocresol (≤25%), alcohol, acid, surfactant, solvent	20	30	1:2000	>4.2

* Due to cytotoxicity of the tested disinfectant the detection limit did not allow detection of higher virus reduction. Abbreviations: IOD = Iodine, CI = confidence interval, MPS = potassium bis (peroxymonosulphate_bis (sulphate), SDIC = sodium dichloroisocyanurate/troclosene sodium, ND = Not determined, QAC = quaternary ammonium compounds, GA = glutaraldehye, DDAC = didecyldimethylammonium chloride, ADBAC = Alkyldimethylbenzylammonium chloride, DDAB = didecyldimethylammonium bromide.

## Data Availability

The data presented in this study are available from the corresponding author. The data are not publicly available due to confidentiality agreements between the manufacturers submitting disinfectants and the testing laboratory.
